# Supplementary material for: Up for grabs: prey herding by penguins facilitates shallow foraging by volant seabirds
Source: R Soc Open Sci. 2019 Jun 5;6(6):190333. doi: 10.1098/rsos.190333 (PMC6599777; doi:10.1098/rsos.190333)
Supplement: Appendix S1 [file rsos190333supp1.docx]

**Appendix S1.**

Animal-borne video recorders (AVR): specifications and programming schedules

Three types of AVRs were used: Replay XD 720 (http://www.replayxd.com) housed in aluminium tubes (dimensions: length X proximal diameter X distal diameter, weight:104 X 26 X 28 mm, 100 g, i.e. <3 % of the average mass of an adult penguin) for 2015 and 2016 deployments; Replay XD 1080 Mini housed in smaller aluminium tubes (dimensions: 94 X 28 X 23 mm, 65 g) for 2017 deployments, and; an Avicam (Zoolog Solutions, South Africa) housed in resin (dimensions: length X width X height: 76 X 33 X 15 mm, 65 g) for one deployment during 2018. AVRs were programmed to record 35 min bins (2015 and 2016) and 15 min bins (2017) with the initial bin set for approximately sunrise and subsequent bins programmed to initiate later in the day. The Avicam was programmed to film continuously from 9h30 until the battery went flat.

Animal-borne video recorder optical calibration

We assessed the optical performance of the Replay XD 1080 by filming a 2D painted model of a sooty shearwater at a range of distances from 5 – 50 m (increments of 5 m) from the camera. A model of a sooty shearwater on the water surface was selected due to it being the smallest species (body length - ca 440 mm [1]), when at the surface, that was regularly observed during this study. Results of the distance image quality calibration are shown in Figure S1. The quality of the images at different distances from the camera can be gauged by a visual assessment of the seabird models with reference to the corresponding number of pixels for each image. The number of pixels were calculated using ImageJ software (ver.1.47,http://imagej.nih.gov/ij) by demarcating a rectangular selection encompassing the extent of the visible seabird model. There was an exponential improvement in image quality at distances < 20 m with the shape of the seabird model being clearly discernible at 5 – 15 m. Although the seabird model can be seen at the full range of distances tested, image quality is noticeably poor at distances > 25 m from the camera.


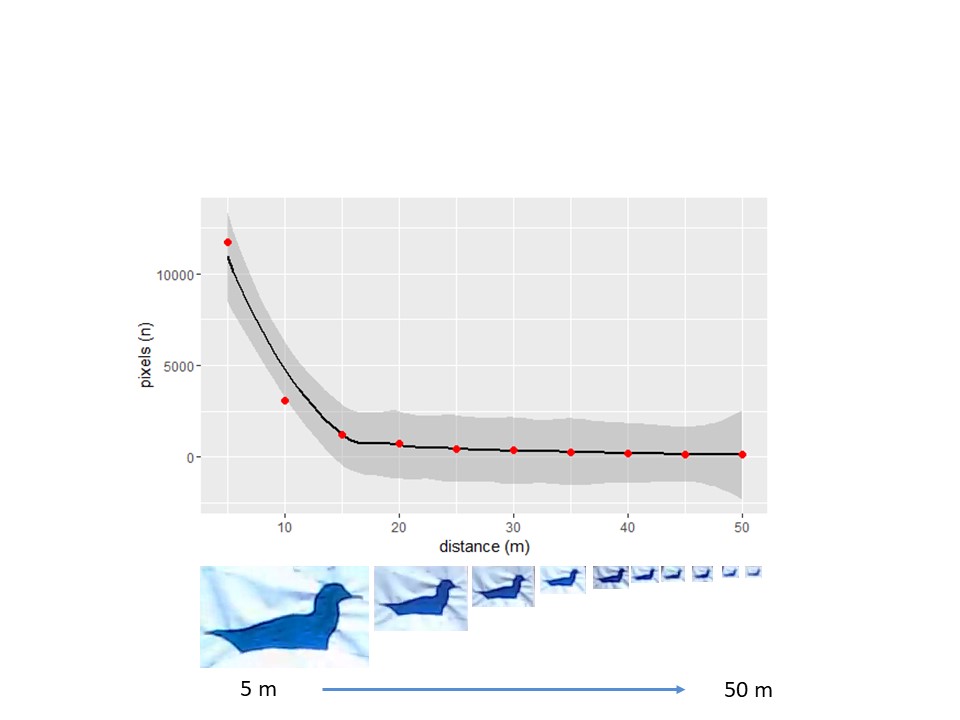


**Figure S1.** Distance calibrated image quality of animal-borne video recorders deployed on African penguins. Top plot represents total number of pixels as a function of each distance increment from the camera. The fitted curve is a loess polynomial regression smooth (family = gaussian, span = 0.75) and shaded areas denote 95 % confidence intervals. The bottom panel contains the actual images of the seabird model at distances ranging from 5 – 50 m (at 5 m increments) from the camera.


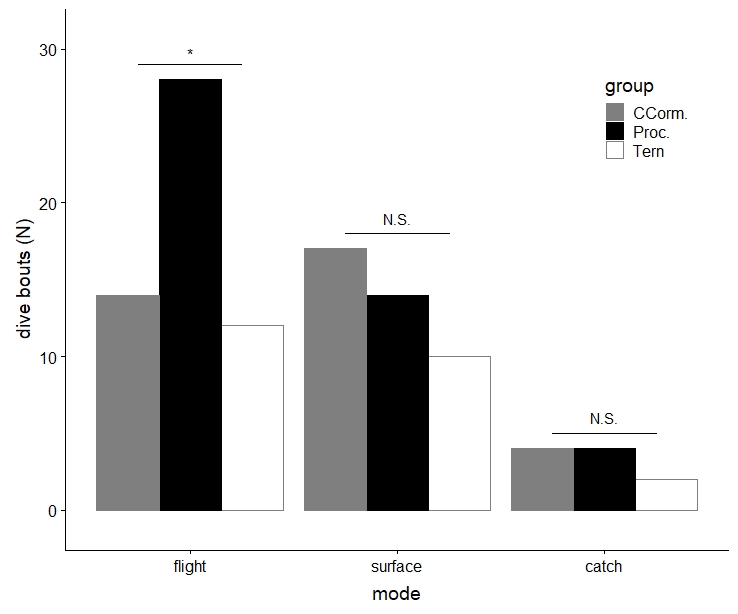


**Figure S2.** Frequency of volant seabird encounters recorded from African penguin AVRs for flight, surface and catch encounters for three species groups, Cape cormorants (CCorm.), Procellariidae species (Proc.) and Terns.

**Table S1.** Summary of Interspecific prey pursuit sequences, elevated schools (ES) and shallow schools (SS).

| **ID** | **pursuit seq.** | **consp (N)** | **school** | | | |  | **interspecific encounter** | | | | **time difference (B-A) s** |
| --- | --- | --- | --- | --- | --- | --- | --- | --- | --- | --- | --- | --- |
|  |  |  |  | **1st encounter** | |  |  | **Cape Corm. (N)** | **Proc. (N)** | **Tern (N)** | **time: 1st prey pursuit (B)** |  |
|  |  |  | **prey** | **time (A)** | **depth** | **catch** |  |  |  |  |  |  |
| SP1601 | SS | 1 | anchovy | 12:11:26 | 3 | 14 |  | 0 | 5 | 0 | 12:11:33 | 7 |
| SP1601 | ES | 6 | anchovy | 07:01:26 | 40 | 16 |  | 3 | 2 | 10 | 07:02:45 | 79 |
| SP1602 | ES | 2 | fish sp. | 06:34:30 | 37 | 7 |  | 5 | 0 | 3 | 06:35:08 | 38 |
| SP1602 | SS | 10 | fish sp. | 11:19:16 | 3 | 4 |  | 1 | 0 | 0 | 11:19:28 | 12 |
| SP1701 | ES | 1 | fish sp. | 09:22:06 | 55 | 12 |  | 2 | 0 | 2 | 09:24:11 | 125 |
| SP1801 | ES | 7 | anchovy | 09:26:59 | 34 | 14 |  | 10 | 7 | 0 | 09:27:41 | 42 |
| SP1801 | ES | 0 | anchovy | 13:39:52 | 49 | 13 |  | 0 | 1 | 0 | 13:41:09 | 17 |
| SP1801 | SS | 2 | anchovy | 14:23:38 | 1 | 14 |  | 4 | 3 | 1 | 14:23:43 | 5 |
| SP1801 | ES | 1 | anchovy | 14:50:49 | 61 | 16 |  | 1 | 4 | 0 | 14:52:37 | 108 |
| SP1801 | ES | 7 | fish sp. | 14:59:40 | 63 | 23 |  | 2 | 7 | 0 | 15:02:14 | 154 |

**References**

1. Ryan PG 2005. Sooty Shearwater. *Puffinus griseus*. In: Hockey PAR, Dean WRJ, Ryan PG (eds) Roberts – Birds of Southern Africa, VII^th^ ed. The Trustees of the John Voelcker Bird Book Fund, Cape Town.
